# Systemic primary carnitine deficiency: an overview of clinical manifestations, diagnosis, and management

**DOI:** 10.1186/1750-1172-7-68

**Published:** 2012-09-18

**Authors:** Pilar L Magoulas, Ayman W El-Hattab

**Affiliations:** 1 Department of Molecular and Human Genetics, Baylor College of Medicine, Houston, TX; 2 Medical Genetics Section, Department of Pediatrics, The Children’s Hospital at King Fahad Medical City and King Saud bin Abdulaziz University for Health Science, Riyadh, Kingdom of Saudi Arabia

## Abstract

Systemic primary carnitine deficiency (CDSP) is an autosomal recessive disorder of carnitine transportation. The clinical manifestations of CDSP can vary widely with respect to age of onset, organ involvement, and severity of symptoms, but are typically characterized by episodes of hypoketotic hypoglycemia, hepatomegaly, elevated transaminases, and hyperammonemia in infants; skeletal myopathy, elevated creatine kinase (CK), and cardiomyopathy in childhood; or cardiomyopathy, arrhythmias, or fatigability in adulthood. The diagnosis can be suspected on newborn screening, but is established by demonstration of low plasma free carnitine concentration (<5 μM, normal 25-50 μM), reduced fibroblast carnitine transport (<10% of controls), and molecular testing of the *SLC22A5* gene. The incidence of CDSP varies depending on ethnicity; however the frequency in the United States is estimated to be approximately 1 in 50,000 individuals based on newborn screening data. CDSP is caused by recessive mutations in the *SLC22A5* gene. This gene encodes organic cation transporter type 2 (OCTN2) which transport carnitine across cell membranes. Over 100 mutations have been reported in this gene with the c.136C > T (p.P46S) mutation being the most frequent mutation identified. CDSP should be differentiated from secondary causes of carnitine deficiency such as various organic acidemias and fatty acid oxidation defects. CDSP is an autosomal recessive condition; therefore the recurrence risk in each pregnancy is 25%. Carrier screening for at-risk individuals and family members should be obtained by performing targeted mutation analysis of the *SLC22A5* gene since plasma carnitine analysis is not a sufficient methodology for determining carrier status. Antenatal diagnosis for pregnancies at increased risk of CDSP is possible by molecular genetic testing of extracted DNA from chorionic villus sampling or amniocentesis if both mutations in *SLC22A5* gene are known. Once the diagnosis of CDSP is established in an individual, an echocardiogram, electrocardiogram, CK concentration, liver transaminanses measurement, and pre-prandial blood sugar levels, should be performed for baseline assessment. Primary treatment involves supplementation of oral levocarnitine (L-carnitine) at a dose of 50–400 mg/kg/day divided into three doses. No formal surveillance guidelines for individuals with CDSP have been established to date, however the following screening recommendations are suggested: annual echocardiogram and electrocardiogram, frequent plasma carnitine levels, and CK and liver transaminases measurement can be considered during acute illness. Adult women with CDSP who are planning to or are pregnant should meet with a metabolic or genetic specialist ideally before conception to discuss management of carnitine levels during pregnancy since carnitine levels are typically lower during pregnancy. The prognosis for individuals with CDSP depends on the age, presentation, and severity of symptoms at the time of diagnosis; however the long-term prognosis is favorable as long as individuals remain on carnitine supplementation.

## Disease name and synonyms

Systemic primary carnitine deficiency

Carnitine deficiency

Systemic primary (CDSP)

Carnitine uptake deficiency (CUD)

Systemic carnitine deficiency (SCD)

Primary carnitine deficiency

Deficiency of plasma-membrane carnitine transporter

## Definition and diagnostic criteria

Systemic primary carnitine deficiency (CDSP) is an autosomal recessive disorder of carnitine transportation typically characterized by episodes of hypoketotic hypoglycemia, hepatomegaly, elevated transaminases, and hyperammonemia in infants; skeletal myopathy, elevated creatine kinase (CK), and cardiomyopathy in childhood; or fatigability in adulthood [1]. There is a considerably broad phenotypic range associated with this condition, ranging from early infantile decompensation to adults who are asymptomatic, therefore, establishing the diagnosis early is essential to guide management. The diagnosis can be suspected on newborn screening, but is established by demonstration of low plasma free carnitine concentration (<5 μM, normal 25-50 μM), reduced fibroblast carnitine transport (<10% of controls), and molecular testing of the *SLC22A5* gene [2].

## Epidemiology

The incidence of CDSP varies depending on ethnicity. In Japan, the incidence of CDSP is approximately 1 in 40,000 [3], whereas the frequency in Australia is approximately 1 in 120,000 [4]. The frequency in the United States and Europe has not been established, however is estimated to occur in approximately 1 in 20,000 - 70,000 individuals based on newborn screening data from various states including Missouri, Texas, and California [personal communication]. Since some individuals with CDSP may remain asymptomatic throughout their entire life, the prevalence of CDSP in the general population may be much higher than originally anticipated since this particular cohort may be underascertained and undiagnosed.

CDSP is an autosomal recessive condition; therefore, we would expect the disorder to occur with equal frequency in males and females. However, apparent sex ratio may be skewed because more adult women may be diagnosed secondary to their infants being diagnosed with CDSP and more adult women than men may actually experience symptoms related to CDSP, particularly during pregnancy which can exacerbate symptoms.

## Clinical description

The clinical manifestations of CDSP can vary widely with respect to age of onset, organ involvement, and severity of symptoms. The most common presentations are in infancy and early childhood with either metabolic decompensation or cardiac and myopathic manifestations, respectively. Adults with CDSP have been reported with mild or no symptoms.

### Infantile metabolic (hepatic) presentation

Approximately half of the reported patients present around the age of 2 years old (range: 3 months – 2.5 years) with metabolic decompensation characterized by episodes of hypoketotic hypoglycemia, hyperammonemia, hepatomegaly, elevated transaminases, and hepatic encephalopathy (poor feeding, irritability, lethargy). These episodes may be triggered by fasting or common illnesses such as upper respiratory tract infections. Cardiomyopathy, skeletal muscle weakness, and mildly elevated creatine kinase (CK) values may be seen in older children with this presentation. Without treatment with intravenous dextrose infusions during these episodes, children may fall into a coma and die [5].

### Childhood myopathic (cardiac) presentation

The remaining half of patients typically present in later childhood around the age of 4 years (range: 1 year to 7 years) with dilated cardiomyopathy, hypotonia, muscle weakness, and elevated CK. Cardiomyopathy in individuals with CDSP can be progressive and may result in death before a diagnosis is established or treatment initiated.

### Adulthood presentation

Adults with CDSP have been reported with no symptoms or mild symptoms including decreased stamina or easy fatigability [1,6-9]. Some women with CDSP have been ascertained after newborn screening identified low carnitine levels in their infants. Carnitine is transferred from the placenta to the fetus during the prenatal period. Therefore, shortly after birth, carnitine levels in newborns reflect those of the mother [10,11]. Expanded newborn screening via tandem mass spectroscopy subsequently detects the low carnitine level in the newborn providing a false-positive result of CDSP for the child. This result should prompt the clinician to probe further into the maternal history and to obtain carnitine levels on the mother since it may be an indication of underlying CDSP in the mother. In studies of women who were ascertained in this way, approximately half were asymptomatic whereas the other half indicated a history of decreased stamina or easy fatigability [1,7-9]. Symptoms, such as decreased stamina or worsening cardiac arrhythmia, may become unmasked during pregnancy [1,8]. This could be due to the increased energy consumption and metabolically challenging state of pregnancy, as well as the normal physiologic decreases in plasma carnitine levels that can occur in any pregnancy [12]. Cardiac findings such as dilated cardiomyopathy and arrhythmias have also been reported in a few adult patients with CDSP [7].

Other, less common, manifestations that can occur in individuals with CDSP include anemia [13], proximal muscle weakness and developmental delay [14], respiratory distress [15], and arrhythmias and electrocardiogram abnormalities [8,16].

## Etiology

Carnitine (3-hydroxy-4-trimethylaminobuyric acid) is mostly derived from dietary intake, but can also be synthesized from lysine and methionine in the liver and kidney. Carnitine is required for the transfer of long-chain fatty acids from the cytoplasm into the mitochondria for beta oxidation [5]. During periods of fasting, fatty acids are the predominant source of energy production. Thus, if carnitine is deficient, this will result in defective fatty acid oxidation. When fat cannot be utilized glucose is consumed without regeneration via gluconeogenesis resulting in hypoglycemia. In addition, fat released from adipose tissue accumulate in the liver, skeletal muscle, and heart resulting in hepatic steatosis and myopathy [2].

CDSP is caused by recessive mutations in the *SLC22A5* gene. This gene encodes organic cation transporter type 2 (OCTN2), which is part of a larger family of OCTNs that play a significant role in transporting organic cation compounds across cell membranes. OCTN2 transfers carnitine across the cell membrane in a sodium-dependent manner. When OCTN2 is not working properly, this can result in improper transfer of carnitine across the cell membrane resulting in a) urinary carnitine wasting leading to low plasma carnitine levels, and b) decreased intracellular carnitine accumulation.

The *SLC22A5* gene is located on chromosome 5q23.3 and is comprised of 10 exons spanning approximately 3.2 kilobases (kb). The gene product, OCTN2, is a transmembrane protein composed of 557 amino acids that includes 12 transmembrane domains and one ATP binding domain. Over 100 mutations have been reported in this gene in the Human Gene Mutation Database (http://www.hgmd.cf.ac.uk/ac/index.php) and the *SLC22A5* database at the ARUP Laboratories (http://www.arup.utah.edu/database/OCTN2/OCTN2_display.php). Approximately half of these mutations are missense, while the other half are composed of nonsense, splice site, and deletion/insertion mutations. These mutations result in dysfunctional OCTN2 gene product and decreased carnitine transport in various tissues. Li et al. sequenced the *SLC22A5* gene in 70 infants with low carnitine levels detected by newborn screening and identified two mutations in 23 infants and one mutation in 25 infants for a detection rate in this population of nearly 70%. The most frequently occurring mutation in this cohort was the c.136C > T (p.P46S) mutation which was identified in about 15% of the mutant alleles [9]. The p.P46S mutation was initially reported in mothers with primary carnitine deficiency identified by expanded newborn screening in the child [8]. Subsequent studies found that the p.P46S mutation is most frequently encountered in asymptomatic or minimally symptomatic adults. The presumption that this mutation may be associated with a milder phenotype was further suggested by studies that demonstrated that the OCTN2 protein product with this mutation retains some residual carnitine transport activity [17].

## Diagnostic methods

### Plasma free carnitine concentration

Measurement of plasma carnitine levels of individuals with CDSP will show extremely reduced plasma free carnitine levels (<5 μM, normal 25-50 μM) [1]. Urine carnitine excretion in individuals with CDSP who are on carnitine supplementation is typically very high. The diagnosis of CDSP may first be suspected when low plasma carnitine concentration is identified via newborn screening using tandem mass spectrometry (i.e. low levels of free carnitine (C0)) [18]. However, since carnitine is transferred across the placenta to the fetus during intrauterine life, shortly after birth, the infants’ carnitine level may actually be a reflection of their mothers’ carnitine levels [10]. This may lead to a false positive diagnosis of CDSP in the infant, but a true positive diagnosis of CDSP in the mother. Carnitine levels in unaffected newborns should normalize within two weeks after birth, therefore, if the initial newborn screen is suggestive of carnitine deficiency, a repeat plasma carnitine analysis should be performed. If the carnitine levels normalize without carnitine supplementation, then there is no additional need to screen the infant for carnitine deficiency.

### Molecular

Further confirmation of the diagnosis, however, relies on molecular genetic testing of the *SLC22A5* gene. Sequence analysis is clinically available and can detect at least one mutation in approximately 70% of affected individuals. Large deletions and duplications of the *SLC22A5* gene, while not a common mechanism of causing CDSP, have been identified in at least one individual with this diagnosis [9]. Therefore, if sequence analysis is negative, or only detects one mutation in an individual strongly suspected of having CDSP, then array comparative genomic hybridization (aCGH) is recommended to screen for larger deletions and duplications that may involve part of or the entire *SLC22A5* gene.

### Fibroblast carnitine transport

If molecular and aCGH fail to detect mutations or large deletions of the gene, then a skin biopsy may be considered to assess carnitine transport in cultured fibroblasts. Carnitine transport in skin fibroblast from individuals with CDSP is typically reduced below 10% of control rates [2,19].

## Differential diagnoses

CDSP should be differentiated from secondary causes of carnitine deficiency. Several groups of inherited metabolic disorders can cause carnitine deficiency, including, organic acidemias and fatty acid oxidation defects such as very long chain acyl-CoA dehydrogenase (VLCAD), medium-chain acyl-CoA dehydrogense (MCAD), long-chain hydroxyacyl-CoA dehydrogenase (LCHAD), and carnitine palmitoyltransferase II (CPT II) deficiencies. CDSP can be differentiated from other fatty acid oxidation defects by demonstration of very low free carnitine levels in plasma (C0) with normal acylcarnitine profile (the concentration of different length acylcarnitine species).

Pharmacological therapy, such as cyclosporine, pivampicillin, and valproate, malnutrition, renal tubular dysfunction, prematurity, and prolonged TPN (total parenteral nutrition) feedings without carnitine supplementation may also cause secondary carnitine deficiency. CDSP can be differentiated from these conditions by careful review of the patient’s medical history, medication history, and biochemical laboratory test results. If plasma carnitine levels are extremely low, and if carnitine levels do not respond to removing the offending agent, then one could pursue further diagnostic testing by sequencing of the *SLC22A5* gene and/or fibroblast carnitine transport assay.

## Genetic counseling

CDSP is inherited in an autosomal recessive manner. Therefore, parents of an individual with CDSP are obligate heterozygous carriers. Heterozygous carriers are asymptomatic. Kozumi et al. reported that left ventricular hypertrophy, described as marginal, late-onset, and benign, was observed in heterozygous mutation carriers of *SLC22A5*[3]. A more recent study however screened a cohort of individuals with cardiomyopathy for mutations in *SLC22A5* and did not see an increased prevalence of heterozygous mutations in this population, concluding that heterozygosity for *SLC22A5* is unlikely to result in a significant risk for cardiomyopathy in adulthood [20]. Heterozygous carriers usually have approximately 50% carnitine transport activity in fibroblasts and may have normal or borderline low carnitine levels in plasma [21]. Therefore, plasma carnitine analysis alone is not sufficient to determine an individual’s carrier status since carnitine levels may be in the normal range. Molecular genetic testing for determining carrier status is considered the preferred method for determining an individual’s carrier status [1].

Since CDSP is an autosomal recessive condition, the recurrence risk in each pregnancy is 25%. Siblings of individuals known to be affected with CDSP should have plasma carnitine analysis to determine if they are also affected with CDSP. However, as mentioned previously, this test is not a reliable method for determining carrier status. In addition, mothers of infants who have an abnormal newborn screen suggestive of carnitine deficiency should have plasma carnitine analysis as well since it may reveal that the mother, rather than the infant, is affected with CDSP since the infant’s carnitine level may be a reflection of the mother’s carnitine level. Repeating the infant’s carnitine level can determine if the infant’s initial low carnitine was a false positive.

Other family members have an increased risk of being a carrier for CDSP. If carrier status needs to be determined, molecular genetic testing is the preferred method and is clinically available. Once the mutations in the family are identified, target mutation analysis can be offered to any at-risk individual or family member interested in determining their carrier status. If one or no mutations are identified in the affected proband, then fibroblast carnitine transport analysis may be offered to screen for carriers since heterozygous carriers have approximately 50% carnitine transport activity when compared to non-carriers.

Genotype-phenotype correlations suggest that nonsense and frameshift mutations are more likely to be associated with lower carnitine transport and are more prevalent in symptomatic individuals whereas missense mutations and inframe deletions many result in residual carnitine transport activity and are more common in asymptomatic individuals [22].

## Antenatal diagnosis

Antenatal diagnosis is possible by molecular genetic testing of extracted DNA from chorionic villus sampling or amniocentesis. However, the identification of both mutations in the *SLC22A5* gene is required in order to be able to offer prenatal diagnosis for this condition. Prenatal diagnosis can also be performed by measuring carnitine transport in amniocytes obtained from amniotic fluid [11]. Antenatal diagnosis for CDSP has seldom been performed since this condition is treatable and carnitine supplementation may be initiated at birth while the diagnostic testing is pending.

## Management

Individuals with the suspected or confirmed diagnosis of CDSP should be referred to a metabolic or genetic specialist so that appropriate management, anticipatory guidance, and genetic counseling can be initiated as soon as possible. Once the diagnosis of CDSP is established in an individual, the following evaluations should be performed for baseline assessment:

Echocardiogram to screen for cardiomyoapthy

Electrocardiogram to screen for arrhythmias

Creatine kinase (CK) concentration to assess muscle involvement

Liver transaminases to assess liver function

Pre-prandial blood sugar to assess hypoglycemia

The metabolic and myopathic manifestations of CDSP can be prevented by maintaining normal plasma carnitine levels. Primary treatment involves supplementation of oral levocarnitine (L-carnitine) at a dose of 50–400 mg/kg/day divided into three doses [2]. The exact dose of carnitine supplementation should be adjusted accordingly based on the individual’s plasma carnitine level. While L-carnitine supplementation has relatively few side effects, high doses may result in increased gastrointestinal motility, diarrhea, intestinal discomfort, and/or the production of trimethylamine, which can result in a fishy odor [19]. Decreasing the carnitine dose may reduce this potential side-effect. If that does not improve the odor, then a course of oral metronidazole at a dose of 10 mg/kg/day for 7–10 days may be indicated.

Maintaining plasma carnitine levels will also reduce the risk of hypoglycemic episodes. Other ways to prevent hypoglycemic episodes in individuals with CDSP would include frequent feeding and avoiding fasting. If an individual with CDSP is hospitalized for hypoglycemia, or needs to fast because of a medical or surgical procedure, treatment with intravenous dextrose is recommended.

To date, there have been no formal surveillance guidelines for individuals with CDSP; however the following screening recommendations are suggested based on the author’s experience (see Table 1):

Echocardiogram and electrocardiogram annually during childhood and less frequently in adulthood. Individuals with cardiomyopathy should be referred to cardiology for further management and treatment.

Plasma carnitine levels should be obtained and monitored frequently (after two weeks of each dose adjustment) until levels reach within the normal range. Once normalization is reached, periodic plasma carnitine analysis should be obtained twice a year in childhood and annually for adults.

CK and liver transaminases measurement can be considered during acute illness.

**Table 1:** Recommended clinical evaluations and management for individuals with systemic primary carnitine deficiency

***Evaluation at time of diagnosis***	***Indication***	***Screening and surveillance***
Echocardiogram and electrocardiogram	Screen for cardiomyopathy and arrhythmias	At time of diagnosis, then annually during childhood; less frequent in adulthood; referral to cardiology if cardiomyopathy or arrhythmia are noted.
Creatine kinase (CK)	Assess muscle involvement	At time of diagnosis, then consider measurement during acute illness
Liver transaminases	Assess liver function	At time of diagnosis, then consider measurement during acute illness
Pre-prandial blood sugar	Assess hypoglycemia	At time of diagnosis, then as needed
Plasma carnitine level	Assess carnitine deficiency and monitor treatment	Obtained and monitored frequently until levels reach within normal range. Then obtain twice a year in childhood; annually in adulthood.
Initiate referrals to genetic or metabolic specialist	Confirmation of diagnosis, molecular genetic testing, genetic counseling, treatment	At time of diagnosis, then twice a year in childhood, annually in adulthood, frequently during pregnancy

Adult women with CDSP who are planning to or are pregnant should meet with a metabolic or genetic specialist ideally before conception to discuss management of carnitine levels during pregnancy. Pregnancy is a metabolically challenging state since energy consumption increases significantly during this period. In addition, women without CDSP typically have lower plasma carnitine levels during pregnancy [12]. Consequences of low plasma carnitine levels in women with CDSP may manifest as increased fatigability or worsening cardiac arrhythmia [1,8]. Therefore, plasma carnitine levels should be closely monitored during pregnancy recognizing that the dose may need to be increased during the pregnancy. Carnitine crosses the placenta during pregnancy and is metabolized to some extent by the placenta [23]; however animal studies and the limited studies on humans have not demonstrated any teratogenic effects on the developing fetus in cases where the mother was supplemented with carnitine at standard doses [24].

## Prognosis

The prognosis for individuals with CDSP depends on the age, presentation, and severity of symptoms at the time of diagnosis. The infantile metabolic and childhood myopathic presentations of CDSP can be fatal if not treated early. Treatment with L-carnitine supplementation should be initiated as soon as possible before irreversible organ damage occurs. In addition, metabolic decompensation and skeletal and cardiac muscle functions improve after carnitine supplementation. The long-term prognosis is favorable as long as individuals remain on carnitine supplementation. Recurrent hypoglycemic attacks and sudden death from cardiac arrhythmia have been reported in several individuals who discontinued carnitine supplementation [2,5,19,25]. These outcomes may have potentially been avoided with proper compliance of carnitine supplementation by the individual and directive and formidable counseling regarding the importance, efficacy, and necessity of prolonged carnitine supplementation by the healthcare provider.

## Unresolved questions

There are reports of individuals with the confirmed diagnosis of CDSP being asymptomatic. The limited literature regarding asymptomatic adults with CDSP and potential health risks make it unclear if treatment in this population is indicated or warranted. However, based on experience with other fatty acid oxidation defects such as medium-chain acyl CoA dehydrogenase (MCAD) deficiency, where individuals can be asymptomatic throughout their life until they have an acute episode during times of stress or illness, and then die suddenly, it has been suggested that carnitine supplementation in asymptomatic individuals with CDSP should be initiated [1]. Diet provides approximately 75% of daily carnitine requirement. This may play a role in modulating the variability and severity of features in individuals with CDSP, especially in those who remain asymptomatic throughout their lifetime.

Ascertainment of individuals with CDSP was initially based on the more severe symptomatic cases. However, with the advent of expanded newborn screening, more infants (and adults) with milder or asymptomatic presentations are getting the diagnosis. Therefore the actual prevalence of this condition in the general population may be much higher than originally anticipated.

## Abbreviations

CDSP: Systemic primary carnitine deficiency; CK: Creatine kinase; OCTN2: Organic cation transporter type 2; aCGH: Array comparative genomic hybridization; VLCAD: Very long chain acyl-CoA dehydrogenase; MCAD: Medium-chain acyl COA dehydrogenase; SCAD: Short-chain acyl-CoA dehydrogenase; LCHAD: Long-chain hydroxyacyl-CoA dehydrogenase; CPT II: Carnitine palmitoyltransferase type II; L-carnitine: Levocarnitine.

## Competing interests

Both authors declare that they have no competing interests.

## Authors’ contributions

Both authors participated in writing this review. They read and approved the final version of the manuscript.
